# Self-Perception and the Relation to Actual Driving Abilities for Individuals With Visual Field Loss

**DOI:** 10.3389/fnhum.2022.852794

**Published:** 2022-03-17

**Authors:** Jan Andersson, Tomas Bro, Timo Lajunen

**Affiliations:** ^1^Swedish National Road and Transport Research Institute (VTI), Linköping, Sweden; ^2^The Eye Unit, Höglandssjukhuset, Eksjö, Sweden; ^3^Department of Psychology, University of Gothenburg, Gothenburg, Sweden

**Keywords:** visual deficits, driving ability, self-perception, driving performance, simulator based driving test

## Abstract

**Background:**

In Sweden, individuals with visual field loss (VFL) have their driving license withdrawn. The literature clearly indicates that individuals with VFL are unsafe drivers on a group level. However, many drivers with VFL can be safe on an individual level. The literature also suggests that self-perception, beliefs, and insights of one’s own capabilities are related to driving performance. This study had three aims: (1) To investigate self-perceived driving capability ratings for individuals with VFL; (2) to compare these ratings between groups with different medical conditions associated with VFL (stroke, glaucoma, and diabetes); and (3) to relate the self-perception ratings to actual driving performance in an advanced driving simulator.

**Methods:**

Participants comprised 723 individuals whose driver’s license had been withdrawn because of VFL and 92 normally sighted elderly individuals. All participants completed a background survey, rated difficulties with different traffic situations, rated their strengths and weaknesses as drivers, and rated aspects that were important for causing traffic accidents. Of the VFL group participants, 264 also completed a simulator-based driving test that they knew could lead to renewal of their driving license. VFL participants and normally sighted was at the same age when they completed the simulator driving test.

**Results:**

Overall, individuals with VFL rated their capabilities as high on all instruments and scales used, even higher than the elderly normally sighted control group. The only VFL etiology group that rated lower than other groups was the diabetes group. Safety orientation and internal control orientation values were best at discriminating between VFL participants in terms of self-perception of driving performance. Participants categorized as “high” in terms of safety skills and internal control were more modest in their ratings. Finally, participants who passed the simulated driving test did not differ from those who failed, in any of the self-perception measures.

**Conclusion:**

Self-perception ratings among individuals with VFL were higher than those of normally sighted elderly individuals. Self-assessed skills did not predict driving performance. Groups with different VFL etiologies rated similarly. Self-ratings of driving abilities cannot be used to assess actual driving performance. Actual driving tests (on road or in the simulator) are necessary to discriminate between safe and unsafe drivers with VFL.

## Introduction

### Drivers With Visual Deficits

Good visual abilities have long been deemed crucial for safe driving behavior (e.g., Wood and Black, 2016). Thus, vision-related requirements for holding a driving license are relatively strict in Sweden (Lindblom, 2011). However, the relationship between visual ability and safe driving is not entirely understood (Owsley and McGwin, 2010). Visual field defects can be a cause for license withdrawal or denial. Such defects are commonly caused by stroke, glaucoma, or diabetic retinopathy.

To hold a driver’s license in Sweden, the extent of the visual field should be at least 120 degrees horizontal and 40 degrees vertical. More than two adjacent missed points with Esterman perimetry within this area precludes driving. No missed points are allowed within the central 20 degrees radius. All corresponding test points within a 10 degree radius must be at least 20 dB, measured with Humphrey perimetry with object size III or equivalent static threshold perimetry. Only one corresponding test point between 10 and 20 degrees is allowed to be less than 10 dB (Transportstyrelsen, 2010, p. 125). Visual defects generally cause a decline in driving abilities; however, this decline does not affect every individual with the same visual defects (Kubler et al., 2014; Blane, 2016; Kwon et al., 2016; Wood et al., 2016). Bowers’ (2016) review article found that it is difficult to predict (using conventional visual field loss (VFL) assessments) which individuals with VFL can drive safely (see also Bro and Andersson, 2021, 2022).

To understand whether perceptual, cognitive, and meta-cognitive abilities were essential for safe driving, Andersson, and Peters (2019) assessed VFL participants’ meta-cognitive ratings and their driving abilities in a driving simulator scenario. Twenty participants with VFL were compared to 83 normally sighted (matched) participants. On a group level, VFL participants rated themselves as “better” compared to the normally sighted participants. No differences were found between the VFL participants who failed and those who passed the simulated driving test.

The current study employed the simulator-based driving test developed by The Swedish National Road and Transport Research Institute (VTI) as an examination assessment tool for individuals with withdrawn driving licenses. The participants who passed the driving simulator test received a renewed driving license if all other medical requirements were fulfilled.

The present study primarily aimed to compare self-perception ratings of driving abilities in normally sighted participants with those of VFL individuals. In addition, the etiology for VFL was addressed; specifically, we investigated whether individuals with stroke, glaucoma, and diabetes differ in terms of their self-perception of driving. We also explored the relationship between a successful driving simulator test result and self-perception ratings for individuals with VFL.

### Older Driver’s Self-Perception

Individuals with VFL are often older. A literature review by Huang et al. (2020) revealed a clear pattern in elderly drivers’ self-perception of driving abilities. The elderly drivers perceived their driving abilities to be better than: their own corresponding abilities at a younger age, their cohorts, i.e., all other drivers. However, as is the case for individuals with VFL, some elderly individuals seem to self-regulate their driving habits according to their driving abilities (Molnar et al., 2014; Ang et al., 2019). As shown by Wood et al. (2013), older individuals will not necessarily be aware of a decline in driving abilities and will adopt appropriate compensatory driving behaviors. A study by Verster and Roth (2012) revealed that subjective assessments do not clearly relate to actual driving performance, either in terms of judgments about alertness before the drive or performance ratings after the driving test.

### Older Drivers With Visual and Cognitive Impairments

In a study by Stalvey and Owsley (2000), over 50% of older individuals with visual impairments did not believe that their vision limitations were likely to cause them to crash. At the same time, approximately 80% believed that avoiding certain specific situations would decrease their crash risk, while 75% actually reported not avoiding those situations. Confidence in driving abilities does not seem to predict driving performance (Riendeau et al., 2016). These studies suggest that the elderly is aware of driving limitations and can even articulate the more problematic situations, depending on the limitation investigated. However, it is unclear whether an understanding of limitations within a specific modality (such as the visual field) can be seen in actual driving behavior, i.e., if there is a relation between driving performance and self-perception. The medical conditions studied were stroke, glaucoma, and diabetes, all of which are associated with VFL.

### Self-Perception of Abilities

Self-perception measures of abilities, beliefs about capabilities, or insights into one’s driving capacity all address what drivers think about the ability in question. Several aspects could be included to capture awareness of the ability, henceforth defined as meta-cognitive abilities. The present study focused on three aspects of meta-cognition and the relation to simulated driving performance. One aspect was difficulties with different traffic situations and experienced vision limitations. Another aspect was individual driving skills, measured with the Driver Skill Inventory (DSI). Finally, drivers’ beliefs related to causes of accidents (T-LoC) can be related to drivers’ likelihood of having an accident.

### Driving Behavior

Driving performance was assessed in terms of self-reported crashes and incidents, and by using a driving simulator test. Self-reported crashes and incidents were included in the assessment as not all participants completed the driving simulator test. Less than 50% of all participants (347 out of 815) were tested and evaluated in the driving simulator (83 out of 92 normally sighted participants, and 264 out of 723 VFL participants). All participants answered the questions about previous crash involvement. The relationship between self-reported crash involvement and a successful driving simulator test result was of interest as well.

The driving simulator test was, as described above, used for the evaluation of driving ability for individuals with VFL. The test result was used for exemption purposes; approximately 200 individuals (see below) without a driving license who were tested have a renewed license today (December 2021). On-road testing is forbidden in Sweden if a VFL is detected. In Sweden, ophthalmologists are obliged to report VFL to the Transportation Agency, which withdraws the license if the requirements for holding it are not fulfilled. The accident involvement follow-up continues. As of December 2021, after 3.8–5.7 years of follow-up (median of 4.6 years of follow-up), 1 of the 200 participants with a renewed license has been involved in a minor traffic accident. The Swedish Traffic Accident Data Acquisition (STRADA) is used for follow-up. The STRADA database contains all reports from the police and the emergency hospitals in Sweden. It is important to mention these points to put the simulated driving test in the correct context. Participants knew that a successful driving simulator test result would give them a renewed driving license. They were highly motivated to perform well. Furthermore, the testing came with a high monetary cost: participants paid approximately €1900 for the test. In addition, the simulator seems to be a valid test of driving abilities. A note of caution is still necessary concerning the validity of simulator-based testing of abilities, given the low number of individuals being followed up and the relatively short period of follow-up.

## Materials and Methods

### Participants

Table 1 shows the number of participants who completed each task. Not all participants completed all tasks. The “other” group comprised individuals with VFL that was not due to stroke, glaucoma, or diabetes. The “unclassified” group referred to individuals who did not reveal their etiology.

**TABLE 1:** Numbers of participants in each VFL etiology group who completed each assessment.

Assessment	Normally sighted individuals (*n*)	VFL etiology participants (*n*)					
		Total	Stroke	Diabetes	Glaucoma	Other	Unclassified
Questionnaire on background, traffic situation difficulties, and self-reported crash involvement	92	723	251	78	203	99	92
DSI*	82	427	132	49	129	62	55
T-LoC scale**	83	412	125	47	128	59	53
Driving simulator test	83	264	108	20	83	24	29
– Passed test		182	72	11	60	18	21
– Failed test		82	36	9	23	6	8

*DSI, Driving skill Inventory; **T-LoC, Traffic Locus of Control.

### Timing of Assessments

Participants completed the questionnaire and scales at different times. The normally sighted individuals answered the DSI and the T-LoC scale before the simulator drive and completed the questionnaire (see Table 1) approximately 4 years after driving simulator test participation. Hence, they were 4 years older when completing the questionnaire. The participants with VFL completed the questionnaire when signing up for the driving simulator test (approximately 36% of individuals with VFL participated in the driving simulator test), and the DSI and T-LoC scale were completed approximately 3 years after the driving simulator test, i.e., the VFL participants were 3 years older at this time. The unsystematic timing of completion was due to results in the Andersson and Peters (2019) study which revealed some interesting findings for the 20 VFL participants on meta-cognition and the data collection was therefore complemented after the simulator test data was analyzed. Even if analyses can be adjusted (by using actual age at the time of completion), this is not optimal, as discussed in the “Results” section.

#### Questionnaire

Twenty-one questions were used to assess: health status, driving experience, driver ability, vision functionality (all on a five-point graded scale); self-reports on incidents and accident involvement; and difficulties with ten different traffic situations (where 1 = not at all difficult and 5 = very difficult).

#### Driver Skill Inventory

The DSI (Lajunen and Summala, 1995; Wallén et al., 2013) contains 20 items and has the following instructions: *“There are many differences between drivers, especially in how we handle different elements when driving. We all have our strong and weak sides. Mark your strong and weak points by filling in the appropriate answer option below.”* The scale consists of 11 items (ranging from 1 = very weak to 5 = very strong) targeting perceptual-motor skills (i.e., quick and fluent car control) and 9 items targeting safety skills (i.e., anticipatory accident avoidance). This instrument was also used to categorize participants as safety-oriented and skill-oriented (see Lajunen and Summala, 1995, for details).

#### Traffic Locus of Control Scale

The T-LoC scale (Özkan and Lajunen, 2005) contains 17 items and gives the following instructions: *“Accidents can be caused by one or more different factors, either because of their own or others’ driving style and the traffic environment. Think of your own driving style and the traffic environment, then estimate how possible you think the different factors below could cause an accident. Mark your answer below.”* Participants were asked to indicate on a five-point scale (where 1 = not at all possible and 5 = highly possible) how possible it is that the 17 listed reasons had caused or would cause an accident. The T-LoC instrument consists of four scales: “Other drivers” (i.e., causes of accidents attributed to other drivers), “self” (i.e., causes of accidents attributed to oneself), “vehicle and environment” (i.e., causes of accidents attributed to external factors), and “fate” (i.e., causes of accidents attributed to fate or bad luck). The instrument also categorized participants based on whether they have an internal or external oriented locus of control (see Özkan and Lajunen, 2005, for details).

#### Simulator-Based Driving Test

The driving simulator test used is described in detail in a paper by Andersson and Peters (2019). The driving simulator (see Nordmark et al., 2004, for technical simulator details) used in this study has a motion system that allows for motion with 4 degrees of freedom and offers both linear and tilt motion. The field of view was approximately 140 degrees (it varies to some extent as a result of the chosen head position). Three LCD displays are used to simulate rear-view and side mirrors. The instructions were as follows. “*You will drive for approximately 50 min and start with a short drive to get used to the simulator. You will drive on rural, highway, and urban roads. Drive as you would in real life.”* All participants were graded by two independent raters in terms of a pass or a fail. One traffic inspector (from the Swedish Transport Administration) and one traffic safety researcher (from the Human Factors department at VTI) conducted the pass/fail grading. Disagreements were rare but were solved by a third rater.

### Statistical Considerations

All ratings were analyzed by analyses of variance (ANOVAs) or multivariate ANOVAs (MANOVAs). Non-parametric analyses were performed as well. When pairwise comparisons were made, Bonferroni corrections were applied. The alpha level of 0.05 was always used.

## Results

The “Results” section is divided into several parts. The first part describes the descriptive statistics for all participants and evaluates the comparability of the participants in the respective groups. However, given the variation in participation for the different scales and the questionnaire, only the data for participants who completed the task or scales analyzed were included, i.e., background data were analyzed for 723 participants with VFL and 92 normally sighted individuals, while the driving simulator test data were only analyzed for participants who completed both the driving simulator test and the questionnaire and scales.

### Background Survey Results

The MANOVA on group (normally sighted vs. five VFL etiology groups) and background survey ratings demonstrated a significant effect for group (see Table 2 for all details). Pairwise comparisons revealed that, generally, normally sighted individuals were older (except for glaucoma participants who were older than all other groups), experienced less good health (except for diabetes participants who rated less good health than all other groups), and had the same functional (corrected if needed) vision (except for diabetes participants who rated lower vision functionality) than all other groups. All VFL groups had a greater need for a driving license, enjoyed driving more (stroke and glaucoma participants rated an even higher enjoyment than diabetes participants), considered themselves better drivers, and rated themselves as better drivers compared to individuals of equal age (except for diabetes participants and those with an etiology classified as “other”). Normally sighted participants were more often involved in serious incidents that were close to, or could develop into, an accident than stroke participants (all pairwise comparisons were corrected for multiple comparisons).

**TABLE 2:** Descriptive statistics (mean values and SD) for background survey data, for normally sighted and all VFL etiology groups.

Parameter	Stroke	Glaucoma	Diabetes	Other	Unclassified	Normal sighted
Patients (*n*)	249	200	78	99	87	92
Age (mean (SD); years)	66.2 (12.57)	73.7 (10.05)	64.6 (9.18)	59.0 (14.25)	67.5 (12.40)	71.2 (5.83)
Health*	1.50 (0.58)	1.33 (0.55)	1.84 (0.74)	1.44 (0.66)	1.40 (0.67)	1.68 (0.67)
Vision*	1.44 (0.60)	1.60 (0.62)	2.01 (0.72)	1.65 (0.70)	1.40 (0.56)	1.53 (0.62)
Need of driving license*	4.51 (0.71)	4.52 (0.64)	4.61 (0.65)	4.45 (0.97)	4.62 (0.74)	4.23 (0.74)
Enjoy driving*	4.56 (0.65)	4.57 (0.61)	4.36 (0.63)	4.55 (0.82)	4.52 (0.75)	4.10 (0.79)
Driving ability*	4.52 (0.66)	4.61 (0.53)	4.49 (0.55)	4.44 (0.77)	4.66 (0.54)	4.23 (0.52)
Ability in relation to those of equal age*	3.87 (0.87)	3.99 (0.79)	3.61 (0.71)	3.75 (0.86)	3.94 (0.94)	3.66 (0.68)
Estimated km/week*	60.47 (240)	113.07 (348)	105.13 (212)	110.91 (234)	111.97 (327)	169.48 (181)
Serious incidents [*n* (%)]	3 (1.2)	9 (4.5)	6 (7)	6 (6)	2 (2)	10 (11)
Accidents with material damage [*n* (%)]	17 (7)	12 (6)	6 (8)	8 (8)	5 (6)	7 (8)
Accidents with personal damage [*n* (%)]	2 (0.8)	2 (1)	0 (0)	2 (2)	0 (0)	0 (0)

*Data represent mean [standard deviation (SD)] subjective ratings.

Subjectively rated crash involvement was tested by Fischer’s exact test. The VFL participants declared less involvement in serious incidents compared to normally sighted participants (*p* < 0.05). Men with VFL reported less involvement in serious incidents than normally sighted men (*p* < 0.05). No other test was significant.

### Difficulties With Various Traffic Situations

The questionnaire contained 10 specific traffic situations but only the most interesting are presented.

The MANOVA for group (normally sighted vs. five VFL etiology groups) and ten traffic situation ratings revealed a main effect of group (see Table 3 for all details). Pairwise comparisons revealed that diabetes participants were the only group to stand out compared to normally sighted participants, i.e., they did not give significantly lower ratings for driving in darkness, during rain, or in unfamiliar areas (even if most ratings for diabetes participants were lower on all traffic situations analyzed) compared to normally sighted participants. Diabetes participants even had a higher rating than normally sighted participants for the driving in darkness traffic situation, although the difference did not reach significance. The only traffic situation that discriminated between the five VFL etiology groups in terms of participant ratings was driving in darkness. The stroke and the unclassified participants rated that driving in darkness was less difficult compared to the glaucoma and diabetes participants (all *p* > 0.05). Participants’ ratings for some of the traffic situations were similar for those with different VFL etiologies; there were no differences between the type of VFL etiology for four of the traffic situations. Table 3 shows the six traffic situations with a significant effect of VFL etiology (the “with passengers” situation was excluded here since the difference were relatively small.

**TABLE 3:** Six traffic situations (mean values (and standard deviations)) with significant effects of VFL etiology and significant pairwise comparisons.

Etiology group	In darkness	Slippery roads	During rain	Crossing without signals	In unfamiliar areas	On high traffic-density roads
Stroke	1.64 (0.76)	1.62 (0.80)	1.36 (0.57)	1.16 (0.42)	1.39 (0.57)	1.27 (0.52)
Glaucoma	2.10 (0.91)	1.50 (0.73)	1.53 (0.69)	1.10 (0.35)	1.41 (0.62)	1.24 (0.53)
Diabetes	2.50 (0.94)	1.49 (0.70)	1.61 (0.69)	1.14 (0.42)	1.53 (0.60)	1.25 (0.54)
Other	1.93 (1.12)	1.56 (0.80)	1.56 (0.84)	1.21 (0.54)	1.46 (0.72)	1.28 (0.64)
Unclassified	1.69 (0.84)	1.53 (0.73)	1.36 (0.60)	1.16 (0.55)	1.33 (0.60)	1.20 (0.55)
Normal sighted	2.35 (0.88)	2.12 (0.81)	1.87 (0.79)	1.39 (0.70)	1.74 (0.72)	1.64 (0.76)

*Data represent mean and standard deviation (in parenthesis).*

### Traffic Locus of Control Scale Analysis

The factor analysis on the T-LoC scale variables revealed the same pattern of results for the VFL participants as for the normally sighted. The “self” factor and the “fate” factor were replicated in line with the model, i.e., the five items on “self” and the three items of “fate” were obtained. However, the “other driver” items and the “vehicle/environment” items loaded on the same factor. None of the items were not loading on a factor. The analysis described below did not include separate items, only using the three main factors of “self,” “fate” and “other.” A high T-LoC scale rating indicates that the factor could be a cause for accidents more often.

**FIGURE 1:**
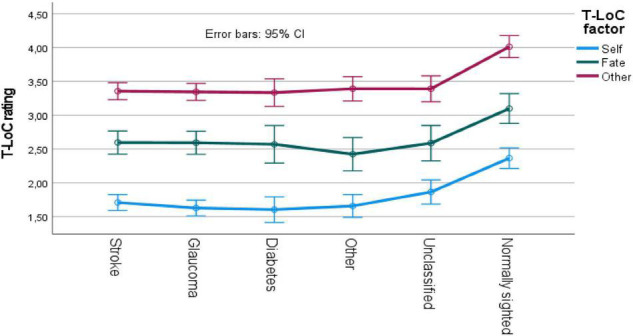
T-LoC scale ratings for VFL and normally sighted participants. CI, confidence interval.

The mixed ANOVA on VFL etiology (six groups) by T-LoC factor (self, fate, and other represents a repeated measurements.) revealed a main effect of VFL etiology [*F*(15, 495) = 15.2, *p* < 0.05, MSe = 1.02] and a main effect of T-LoC factor [*F*(2, 990) = 639, *p* < 0.05, MSe = 0.47]. The interaction effect was not significant. As can be seen in Figure 1, VFL groups rated similarly, and pairwise comparisons revealed that all groups were significantly different compared to normally sighted participants. Pairwise comparisons also revealed that the self-factor were rated significantly lower compared to the “fate” factor, and the “other” T-LoC factor was significantly higher than the “fate” factor (see Figure 1 for details).

### Driver Skill Inventory Analysis

The DSI factors were also well confirmed in our factor analysis, both for VFL and normally sighted participants. Items loaded on the same factors for both groups except for one safety skill item (namely, obeying the traffic lights carefully). The perceptual-motor skill and the safety skill factors were therefore used according to the model without the item that did not load on either of the two factors. Hence, the mean value for the items loaded on each factor on the respective scale were used in the analysis described below. A high value indicates high skill on an item.

**FIGURE 2:**
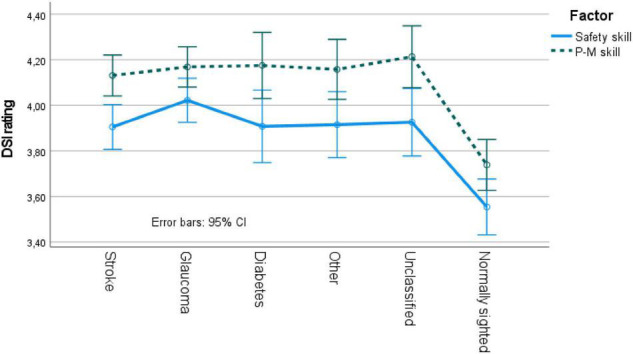
DSI ratings for VFL and normally sighted participants. P-M skill, perceptual-motor skill; CI, confidence interval.

The mixed ANOVA on VFL etiology (six groups) by DSI factor [safety skill and perceptual-motor skill (within-participant variable)] revealed a main effect of group [*F*(5, 482) = 11.0, *p* < 0.05, MSe = 0.42] and a main effect of DSI factor [*F*(1, 482) = 73.4, *p* < 0.05, MSe = 0.15]. The interaction effect was not significant. Pairwise comparisons revealed that safety skills were rated lower than perceptual-motor skills, and that all VFL groups rated higher skills on both factors. No differences were obtained between the VFL participants for either of the factors (see Figure 2).

### Driving Simulator Test Results in the Relation to Self-Perception

The driving simulator test was measured in terms of passed vs. failed. Many parameters were used to evaluate the test drives, conducted by two independent assessors (see Andersson and Peters, 2019; Bro and Andersson, 2021, 2022, for details on test evaluations), but the independent measure used here was passed vs. failed on the simulator-based driving test.

The descriptive analyses (presented below) revealed that 264 of the VFL participants completed the driving simulator test, the traffic situation instrument, and background questions on ability. However, not all of the 264 participants completed the T-LoC scale or the DSI. The degrees of freedom, therefore, vary to some extent between analyses. See Table 4 for the participation numbers of the different etiology groups for the various scales according to pass or failure on the driving simulator test (hence, only the driving simulator tested participants).

**TABLE 4:** Numbers of participants who completed each assessment according to driving simulator test pass or failure.

VFL etiology	Simulator test and questionnaire [*n* (%)]		T-LoC scale (*n*)		DSI (*n*)	
	Passed	Failed	Passed	Failed	Passed	Failed
Stroke	72 (67)	36 (33)	44	19	44	19
Glaucoma	60 (72)	23 (28)	48	17	47	16
Diabetes	11 (55)	9 (45)	9	7	8	6
Other	18 (75)	6 (25)	15	1	14	1
Unclassified	21 (72)	8 (28)	15	6	14	6

**FIGURE 3:**
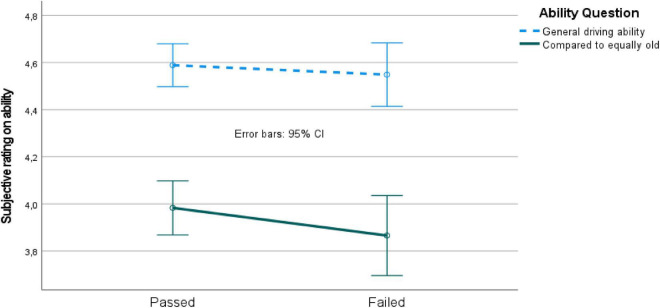
Ability ratings for passed vs. failed driving simulator test participants. CI, confidence interval.

The mixed 2 (passed vs. failed driving simulator test participants) by 2 (ability rating questions; see “Background Survey Results” section above) ANOVA revealed a main effect of ability question [*F*(1, 260) = 139, *p* < 0.05, MSe = 0.34], but no main effect of passed vs. failed, and no interaction effect. The pairwise comparison revealed that the ability questions were significantly separated, i.e., the general ability rating was higher than the ability rating given in relation to those of equal age (Figure 3).

The MANOVA on traffic situations for passed vs. failed driving simulator test participants revealed insignificant effects except for one traffic situation (on pairwise comparison). The passed participants rated that driving “with passengers” was easier than the failed participants [*F*(1, 257) = 7.9, *p* < 0.05, MSe = 0.02].

**FIGURE 4:**
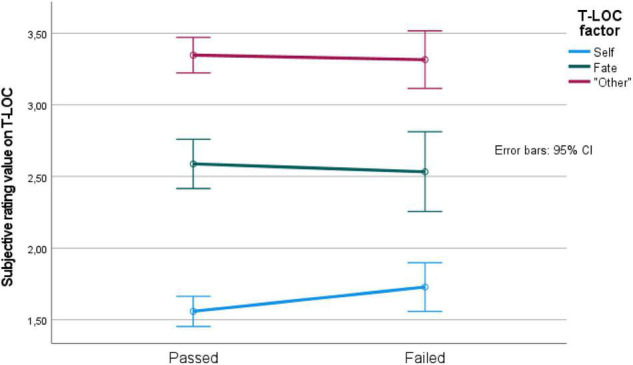
T-LoC factor values for passed vs. failed driving simulator test participants. CI, confidence interval.

The mixed 2 (passed vs. failed participants) by 3 (T-LoC factor) ANOVA revealed a main effect of T-LoC factor [*F*(2, 358) = 241, *p* < 0.05, MSe = 0.43], but no main effect of passed vs. failed participants, and no interaction effect. The pairwise comparison revealed again that the T-LoC factors were significantly separated, as can be seen in Figure 4.

**FIGURE 5:**
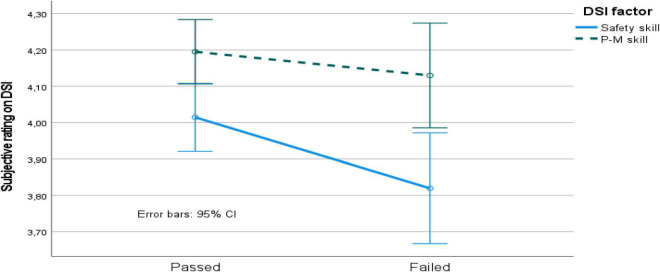
DSI factor values for passed vs. failed driving simulator test participants. P-M skill, perceptual-motor skill; CI, confidence interval.

The mixed 2 (passed vs. failed participants) by 2 (DSI factor) ANOVA revealed a main effect of DSI factor [*F*(2, 173) = 29.3, *p* < 0.05, MSe = 0.14], but no main effect of passed vs. failed participants, and no interaction effect. The pairwise comparison revealed again that the DSI factors were significantly separated. In addition, the participants who passed the driving simulator test rated that their safety skills were better than those who failed (Figure 5).

The separation of participants into different groups based on their VFL etiology in combination with a passed vs. failed classification was only performed for the two larger etiology groups (stroke and glaucoma) for the instruments and scales used (see Table 4). The mixed 2 (passed vs. failed, by 2 (stroke vs. glaucoma) by either (a) ability (2 questions), (b) traffic situation (10 situations), (c) T-LoC factor (3 factors), or d) by DSI (3 factors) ANOVAs revealed no significant effects on passed vs. failed (in all four analyses). Hence no effect of etiology.

The analysis on stated accident involvement (3 different questions; see Table 3) would have contained a very low number of cases and was not performed.

This subsection also considers the driving simulator test results in relation to self-perception, for VFL participants that completed the simulated driving test. The first concern was whether the self-perception of the VFL participants who took part in the driving simulator test differed compared to that of the participants who declined to participate in the simulator. The main reason given for declining to take part was the monetary cost of participation (based on answers given to VTI when a booking time was offered).

The MANOVA on age, gender, health status, need of a driving license, functional vision, enjoyment of driving, driving ability in general, and driving ability compared to individuals of equal age, revealed that those VFL participants who took part in the driving simulator test stated a higher need for a driving license [*F*(1, 707) = 5.08, *p* < 0.05, MSe = 0.53], a better health status [*F*(1, 707) = 5.14, *p* < 0.05, MSe = 0.40], and better functional vision [*F*(1, 707) = 7.02, *p* < 0.05, MSe = 0.42]. However, no differences were obtained in terms of the enjoyment of driving a car, age, and, most importantly, driving ability ratings (two questions) or incident or crash involvement (all *p* > 0.05).

### Safety- vs. Skill-Oriented and Internal vs. External Oriented Control

Finally, to investigate how self-perception was related to driving ability, participants were divided into three unique categories (high, medium, and low) based on their DSI and T-LoC scale ratings, respectively. For example, DSI values were categorized into high, medium, or low on skill orientation (the participants’ skill orientation score was subtracted by the safety orientation score). The size of categories was divided based on the distribution of values, i.e., a relative even proportion of participants were categorized as high, medium, and low rather pragmatically (see Table 5 for details). Participants were generally more external control oriented (T-LoC scale) and more skill-oriented (DSI), as the mean values used for classification in Table 5 reveal.

**TABLE 5:** Numbers of VFL participants in each DSI and T-LoC scale ratings category.

Safety orientation	External oriented (T-LoC scale)			
	High (4.0–2.0)	Medium (1.99–1.48)	Low (1.47—1.47)	Total number
High (−1.60 to 0.00)	49	33	51	133
Medium (0.01–0.30)	10	23	43	106
Low (0.31–2.12)	50	44	74	168
Total	139	100	168	407

*Data in parentheses represent the mean inclusion values for each ratings category.*

The analyses described below only used the high and low ratings categories to maximize the differences between groups, i.e., high safety–high external, high safety–low external, low safety–high external, and low safety–low external.

The first MANOVA conducted included the four ratings categories as a between-participant variable and the following background question variables as dependent measures: age, health, vision, need of a driving license, kilometers driven, enjoyment of driving, general driving ability, and driving ability compared to those of equal age. This revealed significant effects for: the need of a driving license [*F*(3, 214) = 4.56, *p* < 0.05, MSe = 0.42], enjoyment of driving [*F*(3, 214) = 4.60, *p* < 0.05, MSe = 0.45], general driving ability [*F*(3, 214) = 9.85, *p* < 0.05, MSe = 0.25], and driving ability compared to those of equal age [*F*(3, 214) = 9.13, *p* < 0.05, MSe = 0.64]. Pairwise comparisons revealed that participants in the category of high safety orientation/high internal control orientation (low external) rated differently compared to the other three categories (who rated similarly to each other). They had less need of a driving license, enjoyed driving less, reported lower general driving abilities, and reported being less capable compared to those of equal age. Even if the values for the category of high safety orientation/high internal control orientation were lower on these four background questions, the mean values were still high.

The second MANOVA with the four ratings categories and different traffic situations as the dependent measures (see Table 4 for details) revealed that slippery roads [*F*(3, 212) = 4.98, *p* < 0.05, MSe = 0.53], driving in unfamiliar areas [*F*(3, 212) = 3.10, *p* < 0.05, MSe = 0.31], and driving on high traffic-density roads [*F*(3, 212) = 4.24, *p* < 0.05, MSe = 0.24] were significant. Pairwise comparisons revealed again that the category of high safety orientation/high internal control (low external) stood out. The participants found these three traffic situations more difficult to perform (again, relatively speaking).

Finally, the four ratings categories were studied in terms of passes or fails on the driving simulator test. As shown in Table 6, the number of participants was considerably reduced. The chi-square comparison revealed an insignificant effect. The orientation given by the DSI, or T-LoC scale values could not be related to a pass or a fail in the simulator-based test.

**TABLE 6:** Numbers of participants for the four ratings categories according to driving simulator test results.

Simulator test result	High safety/high internal	High safety/high external	Low safety/high internal	Low safety/high external	Total number
Passed	13	21	16	23	73
Failed	4	4	4	9	21
Total	17	25	20	32	94

The VFL etiology was also analyzed in terms of the four categories studied. The analysis revealed that the numbers of participants with each VFL etiology were evenly distributed across categories.

## Discussion

### Comparison of Visual Field Loss With Normally Sighted Participants

In Sweden, individuals with VFL have their driving license withdrawn if the specified requirements for holding a license are not fulfilled (see Commission Directive 2009/113/EC, for details). The frustration of the individual’s experience of this is well documented (Nyberg et al., 2019).

The results showed that the ratings of VFL participants who took part in the simulator-based driving test differed to those of the VFL participants who did not take part, although no differences were found for ability ratings or stated crash involvement. Those VFL participants who participated in the driving simulator test expressed a greater need for a driving license and reported better health and functional vision. The results suggest that the decision to participate in the driving simulator test (which cost each individual nearly €1900) was mostly based on a greater need for a driving license and their financial situation. However, the two groups (driving simulator participators and non-participators) within the VFL group did not believe that their driving abilities differed. The following discussion therefore considers the VFL group as one group.

The overall results from the analysis of the background questions revealed that VFL participants were more motivated car drivers and perceive that they have better functions and the abilities needed to drive safely, compared to normally sighted individuals. The VFL participants even believed that they generally drive better, especially men, and they had not been involved in serious incidents to the same extent as the normally sighted participants. Based on the results, the frustration expressed in the different VFL etiology groups studied is unsurprising and is in line with the findings of previous studies (e.g., Nyberg et al., 2021).

### Differences Between Visual Field Loss Etiology Groups

The background analysis revealed that most VFL etiology groups rated similarly to each other. The diabetes group was the only group that stood out to some extent. Diabetes participants reported less good health and worse vision functionality. However, although diabetes participants’ ratings of their abilities were not as high as for stroke and glaucoma participants, they were as high as the normally sighted participants’ ratings.

The diabetes participants rated a few of the traffic situations studied differently. Driving in dark areas in particular was experienced as more difficult. However, the overall results indicated that the VFL etiology groups’ ratings were very similar (except for the diabetes group to some extent), and the traffic situations tested were experienced as relatively easy to drive in.

When accident-causing factors (T-LoC scale) were studied, VFL etiology seemed to be unrelated to the obtained results. All VFL etiology groups rated all factors lower than the normally sighted individuals. The results were clear, with considerable similarities between the VFL etiology groups. The same was true when driver skill (measured using the DSI) was studied: the VFL etiology groups’ ratings were similar on all factors and were significantly higher than the normally sighted individuals’ ratings.

### Differences Between Safety/Skill and Internal/External Control Orientation

Participants with VFL had high ratings, with relatively low variation. However, when the four rating-based categorizations were used to create different groups, some interesting differences were obtained. When self-perception was measured by questions related to the participants themselves, such as the need for a driving license and their beliefs about their own driving abilities, one of the four categories was rating differently compared to the other three categories; the participants with the highest ratings toward a safety orientation in combination with the highest ratings on internal control orientation were more modest in their ratings. The same pattern was obtained for difficulties with different traffic situations, but it was not as clear.

However, the categorization used could not predict the outcome of the driving simulator test. In addition, VFL etiology was evenly distributed across the ratings categories. The diabetes participants revealed some modesty in ratings of their abilities and performance in traffic situations; however, they were not oriented differently compared to the other VFL etiology groups. It should be noted that the classification was somewhat arbitrary and driven by an idea of even distribution for the groups. It could not discriminate between safe and unsafe drivers in the simulator test. Hence, participants categorized as high/low on safety orientation and high/low on internal control are not necessarily safe drivers. The results should be interpreted with caution.

### Simulator Driving Performance

Only VFL participants were assessed with the driving simulator test in terms of a pass and fail. Data from normally sighted participants’ driving performances were used as reference values for different driving simulator test events in the evaluation of VFL participants. The normally sighted individuals were unfortunately not classified into the categories of pass or fail, because of the limited availability of traffic inspectors.

The VFL participants’ test results were compared to the reference groups’ values on time-to-collision and time headways, collision, and so on. Bro and Andersson (2021) showed that glaucoma participants who failed (compared to glaucoma participants that passed) the tests had lower performance measures on several safety margins studied.

The overall results revealed that the ratings on the instruments and scales used were unrelated to driving performance. When the largest VFL etiology groups were analyzed, the same pattern emerged, i.e., there were no differences in ratings on the instruments or scales used, for the participants who passed or failed.

Anstey et al. (2005) suggested that cognition, sensory function, and physical function or medical conditions were factors enabling safe driving. Accurate self-monitoring of these factors was required for safe driving. The present schematic model of factors enabling safe driving behavior describes how: (1) Cognition, vision, and physical function are related to the capacity to drive safely; (2) cognition is related to self-monitoring and beliefs about driving capacity; and (3) self-monitoring, and beliefs about driving capacity and capacity to drive safely, are related to driving behavior. Hence, the model separates driving behavior from the capacity to drive safely and shows that insights into one’s driving capacity are a secondary factor influencing actual driving behavior. The results obtained in this study do not support this model in a sufficient way. It could not be seen in the results obtained that self-monitoring (in terms of self-perception ratings) could discriminate between safe vs. unsafe drivers. In the Andersson and Peters (2019) study main effects of cognition performance was obtained between VFL groups and normally sighted on a group level. However, cognitive results could not discriminate between safe vs. unsafe drivers, except for reaction time measures that was context dependent. Thus, the perceptual capabilities on reaction time tasks that was not context dependent (measured by a classical Simon task) did not discriminate between safe vs. unsafe drivers.

The only reasonable explanation for all these results is that driving ability is really context dependent and extremely difficult to predict with more or less sophisticated instruments and classical testing. It could be the case that self-perception and self-monitoring affect the strategic and tactical levels. Meaning, the decision to stop driving or avoid some situations might be affected by some self-regulation mechanisms. But performances on an operative level, i.e., drive the car in a safe manner, is not related to self-perception.

### Study Limitations

This study has two noteworthy limitations. First, and most importantly, the VFL population in Sweden is frustrated by the driving-related requirements. It is difficult to fully understand and explain political decisions, and it is beyond the remit of this study to attempt to do so. Nevertheless, in June 2020, the Swedish parliament decided to address the situation. The Swedish government initiated an inquiry into the matter and tasked the governmental investigator with finding a solution for measuring driving ability that did use only perimetry as a reason for dispensation. The data collected in the current study were obtained during a period of participant frustration. The VFL participants were drawn from a pool of individuals highly motivated to take part in a driving ability experiment. However, no differences were obtained between those VFL participants who passed and those who failed the driving simulator test. Neither was there a difference between the participants who took part in the driving simulator test and those who declined.

The second limitation is that not all participants completed all the instruments and scales at the same time. Several participants completed the T-LoC scale and the DSI after receiving the driving simulator test results, which could have affected the participants’ answers. Nevertheless, as stated previously, there were no differences in the ratings of the participants who passed and failed. Although it is believed that these two limitations did not affect the results, their influence cannot be ruled out.

## Conclusion

The present study aimed to investigate whether self-perception of driving abilities in participants with VFL is related to actual driving performance or VFL etiology. The main conclusion is that such self-perception is not related to actual driving performance, as measured here by different meta-cognitive scales and a simulator-based driving test. The diabetes group was the only VFL etiology group to rate themselves differently compared to the other groups but only to a small extent.

The tasks performed in this study are explicit by nature, and the self-monitoring or beliefs about driving capacity, as discussed by Anstey et al. (2005), might be driven by implicit reasoning as well. However, the beliefs and insights about driving capacity, as measured here, were not related to actual driving ability. The conclusion of Huang et al. (2020), whereby the elderly perceives themselves to be better drivers than themselves at a younger age, their cohorts, i.e., all other drivers, was supported by this study, and hence appears to be true for individuals with VFL as well.

The results also indicated that participants with a more safety-oriented and internal control orientation were more modest about their abilities, generally speaking, even if it was not possible to discriminate between safe and unsafe drivers in the simulated driving test. This is interesting and should be investigated further.

As Wood et al. (2013) suggest, it should *not* be assumed that when older individuals’ driving abilities begin to decline, they will necessarily be aware of these changes and adopt appropriate compensatory driving behaviors. This was supported for individuals with VFL, although diabetes participants were more aware of a decline in functional vision and health associated with the VFL etiology.

It can also be concluded, as the Verster and Roth (2012) study revealed, that subjective assessments do not clearly relate to actual driving performance or ratings of performance after the simulated driving test. Among individuals with VFL, ratings of self-perceived abilities are high, even higher than ratings of normally sighted elderly individuals, even if the scales were completed after performance in the driving simulator test. The self-perception ratings cannot predict driving performance. All VFL etiology groups rated very similarly. Self-perception or beliefs about driving abilities can therefore not be used as an accurate assessment of driving performance. An actual driving test is still necessary to discriminate between safe and unsafe drivers with VFL.

## Data Availability Statement

The raw data supporting the conclusions of this article will be made available by the authors, without undue reservation.

## Ethics Statement

The studies involving human participants were reviewed and approved by the Linköping University Committee (Dnr 2014/124-31). 2.2. The patients/participants provided their written informed consent to participate in this study.

## Author Contributions

JA was the main author. TB and TL worked with the manuscript. All authors contributed to the article and approved the submitted version.

## Conflict of Interest

The authors declare that the research was conducted in the absence of any commercial or financial relationships that could be construed as a potential conflict of interest.

## Publisher’s Note

All claims expressed in this article are solely those of the authors and do not necessarily represent those of their affiliated organizations, or those of the publisher, the editors and the reviewers. Any product that may be evaluated in this article, or claim that may be made by its manufacturer, is not guaranteed or endorsed by the publisher.
